# Lean mass as a determinant of bone mineral density of proximal femur in postmenopausal women

**DOI:** 10.20945/2359-3997000000059

**Published:** 2018-08-01

**Authors:** Rosangela Villa Marin-Mio, Linda Denise Fernandes Moreira, Marília Camargo, Neide Alessandra Sansão Périgo, Maysa Seabra Cerondoglo, Marise Lazaretti-Castro

**Affiliations:** 1 Universidade Federal de São Paulo Universidade Federal de São Paulo Disciplina de Endocrinologia São Paulo SP Brasil Disciplina de Endocrinologia da Universidade Federal de São Paulo (Unifesp), São Paulo, SP Brasil; 2 Universidade Federal de São Paulo Universidade Federal de São Paulo Disciplina de Geriatria e Gerontologia São Paulo SP Brasil Disciplina de Geriatria e Gerontologia da Universidade Federal de São Paulo (Unifesp), São Paulo, SP, Brasil

**Keywords:** Osteoporosis, treatment, body weight, body composition

## Abstract

**Objective:**

To verify which component of body composition (BC) has greater influence on postmenopausal women bone mineral density (BMD).

**Subjects and methods:**

Four hundred and thirty women undergoing treatment for osteoporosis and 513 untreated women, except for calcium and vitamin D. Multiple linear regression analysis was performed in order to correlated BMD at lumbar spine (LS), total femur (FT), femoral neck (FN) with body mass (BM), total lean mass (LM) and total fat mass (FM), all determined by DXA.

**Results:**

BM significantly correlated with all bone sites in untreated and treated women (r = 0.420 vs 0.277 at LS; r = 0.490 vs 0.418 at FN, r = 0.496 vs 0.414 at FT, respectively). In untreated women, the LM correlated better than FM with all sites, explaining 179% of LS; 32.3% of FN and 30.2% of FT; whereas FM explained 13.2% of LS; 277% of FN, 23.4% of FT In treated women, correlations with BC were less relevant, with the LM explaining 6.7% of BMD at LS; 15.2% of FN, 16% of FT, whereas the FM explained 8.1% of LS; 179% of FN and 176% of FT.

**Conclusion:**

LM in untreated women was better predictor of BMD than FM, especialy for distal femur, where it explained more than 30% of the BMD, suggesting that maintaining a healthy muscle mass may contribute to decrease osteoporosis risk. Treatment with anti-osteoporotic drugs seems to mask these relationships. Arch Endocrinol Metab. 2018;62(4):431-7

## INTRODUCTION

Osteoporosis is intimaly associated to the aging process and represents a social problem nowadays. Populational statistics shows that the situation can get even worse in the future due to the increase in longevity. Low bone mass is one of the main determinants of osteoporosis, which associates with bone microstructural changes resulting in a higher fracture risk. By comprehending what positively influences bone mass of individual health professionals will be able to create strategies to control bone loss throughout aging (1,2).

Total body mass is one of the biological variables that best correlates with bone mass. However, it remains unclear what would be the influence of the different body mass components on bone metabolism (1,3–6). Gillette-Guyonnet and cols. (5) studied older osteoporotic women (75 to 89 years old) and observed a significant correlation between bone mineral density and body composition (BC), which includes body mass, fat mass and lean mass. In this study, fat mass showed a better association with bone mass than lean mass, suggesting that fat mass could exert a protective effect on the proximal femur. On the other hand, Binder and Kohrt (7), studying elderly men and women, observed that the lean mass was the BC component that best correlated with bone mineral density (BMD). The authors suggested that the association of lean mass and fat mass with bone mass reflects not only the effects of total body mass mechanical loading on bone, but also the functional relation between muscles and bones.

It is believed that muscle contractions, as well as physical exercise, act as potent anabolic stimuli to strengthen bone tissue. On the other hand, low fat mass can represent especially relevant state of denutrition in elderly, which could reflect on the health of bone tissue, currently recognized as a tissue involved in energy metabolism (2,8).

As studies about BC and bone mass still show controversial results, our aim was to determine which of the components of BC would be better related to bone mass in a representative population of postmenopausal women both treatment and treatment naive for osteoporosis.

## SUBJECTS AND METHODS

The sample consisted of 943 independent postmenopausal women, with age above 40 years old (average 66.9 ± 7.8 years old), who volunteered to participate in three different protocols to evaluate physical exercise effects on bone mass, conducted by the same group of researchers. The present crosssectional study used the baseline data from all these women as they entered the study protocols, which were conducted by the same professionals and utilized the same methodology for measurement of antrhopometric and body composition parameteres. The selection criteria for choosing study participants are described in details in the publications resulting from these three studies (9–11). For further analysis, these women were divided into two groups: 430 that were undergoing treatment for osteoporosis (47 to 87 years old, average of 68.3 ± 8.2 years old) (10,11) and 513 never treated for osteoporosis (41 to 87 years old, average of 65.8 ± 7.4 years old) (9,11).

The study has the approval of the Ethics and Research Committee of *Universidade Federal de São Paulo* - Unifesp/EPM, numbered CEP: 32882/12, CAAE: 02252312.1.0000.5505. All the subjects signed an informed consent.

The methods selected to evaluate the total and the segmental BC, as well as the total and the compartmental anthropometric measurements and sites of bone mineral density are described as follows.

The total body mass was measured using a platform-type mechanical scale (Filizola, São Paulo, Brazil) with a maximum capacity of 150 kg and variation 0.1 kg. Height was measured using a vertical bar stadiometer with maximum range of 220 cm and accuracy of 0.1 cm. Body mass index - BMI (kg/m^2^), was calculated from weight and height measurements using the formula BMI = weight (in kg) divided by height (in m^-2^) (12).

The BC and the bone mineral density (BMD) analyzes were obtained by dual-energy X-ray absorptiometry (DXA), in a Hologic QDR 4500A equipment (Waltham, MA). This assessment was performed at the Bone Evaluation Laboratory of the Endocrinology Division, at Universidade Federal de Sao Paulo. In our hands the CV% for lumbar spine and total femur is 1%, for trochanter is 1.2% and for femoral neck is 1.4%. The studied variables were: BMD in the sites of lumbar spine L1-L4 (LS), total femur (TF) and femoral neck (FN) in grams/cm^2^ and the T-score values; in addition to the lean mass (LM) and fat mass (FM) in absolute values.

### Statistical analysis

Normality of the data was assessed by using the Kolmogorov-Smirnov adherence test. When the groups were divided into treated and untreated women, they were compared by the “t” test of Student for independent samples in order to determine whether the groups presented any different characteristics.

The Pearson Linear correlation, as well as the univariate linear regression and the analysis of multiple linear regressions were performed, having the bone sites as the dependent variables and BC (total body mass, total lean mass) as the independent ones.

The studied variables that presented p < 0.20 in the Pearson linear correlation analysis were selected and included in the models and, further, considered for inclusion in the multiple linear regression model. For this model, the stepwise forward modeling strategy was used.

The variables that remained significant were kept in the final multiple linear regression model, always observing the possible collinearities. The variable age was considered as a control variable. All the analisys were made by using the Statistical Package for the Social Sciences - SPSS for Windows - version 19”.

## RESULTS

All studied variables were different between the two evaluated groups - women treated and untreated for osteoporosis, emphasizing that the treated participants were older, thinner, shorter, presented a worse bone mass and lower values of body composition components (Table 1).

**Table 1:** Descriptive characteristics of bone mass and body composition of untreated and treated women for osteoporosis

Variables	Untreated n = 513				Treated n = 430			
	x	sd	min	max	x	sd	min	Max
Age (years)	65.8*	7.4	41	87	68.3	8.2	47	87
Weight (kg)	70.8*	13.4	36.0	111.6	60.7	11.3	35.0	104.0
Height (m)	1.55*	0.06	1.36	1.90	1.53	0.07	1.33	1.74
BMI (kg/m^2^)	29.5*	5.1	14.4	50.1	25.9	4.2	16.6	42.7
BMD LS (g/cm^2^)	0.933*	0.156	0.464	1.608	0.759	0.128	0.371	1.634
BMD FN (g/cm^2^)	0.764*	0.128	0.398	1.342	0.657	0.097	0.408	1.010
BMD TF (g/cm^2^)	0.878*	0.126	0.449	1.266	0.756	0.108	0.350	1.052
T score LS	− 1.0*	1.4	− 5.3	5.1	− 2.6	1.1	− 6.1	5.3
T score FN	− 0.8*	1.1	− 4.1	4.4	− 1.7	0.9	− 4.0	1.5
T Score TF	− 0.5*	1.1	− 4.0	2.7	− 1.5	0.9	− 4.9	0.9
Lean mass (kg)	42.1*	6.1	27.9	59.8	37.3	5.1	24.4	67.0
Fat mass (kg)	27.5*	8.1	9.5	50.9	21.9	6.9	7.5	48.5

* p < 0.05 treated vs untreated.

**Figure 1:**
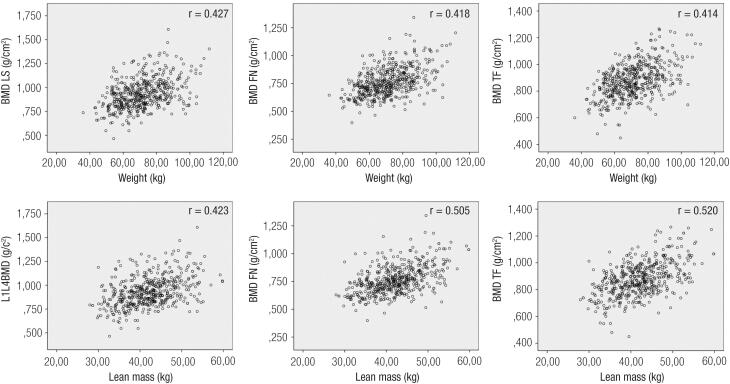
Correlation charts between the bone sites, total body mass and lean mass of untreated women for osteoporosis.

In women not treated for osteoporosis, among all the studied variables, the total body mass was the one that best correlated with all sites of bone mass: LS r = 0.427 (p = 0.000), FN r = 0.490 (p = 0.000) and TF r = 0.496 (p = 0.000). The correlation between the different sites of bone mass with height or BMI showed values ranging from r = 0.120 to r = 0.430 (p = 0.000). For the same 513 women, the lean mass was the variable that best correlated with all sites of bone mineral density, r = 0.423 (p = 0.000) at LS, r = 0.505 (p = 0.000) at FN and r = 0.520 (p = 0.000) at TF (Figure 1). The associations with fat mass were also significant but showed less expressive results in all sites [r = 0.361 (p = 0.000) for LS; r = 0.433 (p = 0.000) for FN and r = 0.430 (p = 0.000) for TF].

After performing the individual analyzes, we started to build statistical models to determine the influence of lean and fat mass on bone mass in the 513 untreated women and the 430 women treated for osteoporosis.

In untreated women, the coefficients of BMD determination found for lean mass models were better than the ones found for the fat mass: 17.9% in BMD of LS; 32.3% for FN and 30.2% of TF. On the other hand, models showed that fat mass could explain 13.2% of LS BMD; 27.7% of FN BMD and 23.4% of TF BMD (Table 2).

**Table 2:** Results of the multiple linear regression analysis between the sites of BMD and lean body mass and fat mass variables of 513 untreated women for osteoporosis

Independent variables	Lumbar spine (g)		Femural neck (g)		Total femur (g)	
	β	p	β	p	β	p
Model lean mass						
Constant	0.480	0.000	0.669	0.000	0.659	0.000
Lean mass (kg)	0.011	0.000	0.009	0.000	0.010	0.000
Age (years)*	− 4.508	0.996	− 0.005	0.000	− 0.003	0.000
r	0.423		0.558		0.549	
r^2^	0.179		0.323		0.302	
p	0.000		0.000		0.000	
Model fat mass						
Constant	0.795	0.000	0.936	0.000	0.956	0.000
Fat mass (kg)	0.007	0.000	0.006	0.000	0.006	0.000
Age (years)*	− 0.001	0.389	− 0.005	0.009	− 0.004	0.000
r	0.363		0.526		0.483	
r^2^	0.132		0.277		0.234	
p	0.000		0.000		0.000	

* All adjustments for age.

In women treated with active drugs for osteoporosis, the correlation between BMD and body mass became weaker. In this group, the models for lean mass explained only 6.7% of LS BMD; 15.2% of FN BMD and 16% of TF BMD (Table 3), while the model for fat mass explained 8.1% of LS BMD, 17.9% of FN BMD and 17.6% of FT BMD. Therefore, treatment of osteoporosis appears to modify the relation between bone mass and the anthropometric variables, decreasing the great influence of these parameters on the BMD.

**Table 3:** Results of the multiple linear regression analysis between the sites of BMD and lean body mass and fat mass variables of 430 treated women for osteoporosis

Independent variables	Lumbar spine (g)		Femural neck (g)		Total femur (g)	
	β	p	β	p	β	p
Model lean mass						
Constant	0.453	0.000	0.498	0.000	0.637	0.000
Lean mass (kg)	0.006	0.000	0.007	0.000	0.007	0.000
Age (years)*	0.001	0.178	− 0.001	0.010	− 0.002	0.000
r	0.258		0.390		0.399	
r^2^	0.067		0.152		0.160	
p	0.000		0.000		0.000	
Model fat mass						
Constant	0,577	0.000	0.632	0.000	0.786	0.000
Fat mass (kg)	0.005	0.000	0.006	0.000	0.006	0.000
Age (years)*	0.001	0.191	− 0.001	0.009	− 0.002	0.000
r	0.285		0.423		0.419	
r^2^	0.081		0.179		0.176	
p	0.000		0.000		0.000	

* All adjustments for age.

## DISCUSSION

The total body mass in adults is one of the biological variables that most consistently correlate with bone mass and fracture risk (8). In our study, this phenomenon was detected with greater relevance among women without treatment for osteoporosis. Treatment with specific drugs for osteoporosis made the relationship between BMD and body mass less relevant, probably because these drugs directly interfere on bone remodeling, mitigating the local influence of body mass (2). In a similar study with Italian Caucasian postmenopausal women, authors (13) concluded that both fat and lean masses might affect bone mass but depending on the osteoporotic status. In non-osteoporotic women, only lean mass was associated with BMD. In osteoporotic women treated for osteoporosis, however, the lean and fat masses had the same importance.

Lewin and cols. (14) also studied a population of Brazilian Caucasian women and observed that heavier girls reached bone mass peak earlier, besides having higher BMD values. In addition, the bone loss caused by aging was reduced in those women with higher total body mass.

In our study, the correlation of total body mass was more relevant with the proximal femur than with the lumbar spine, what could translate influence of the mechanical load and physical activity on bone mass of lower limbs. To contribute to this theory, in our results the lean mass was the component that best correlated with bone density in different places, especially the proximal femur.

Reid (3), in a review study, emphasizes that total body mass is a main determinant of bone mineral density as well as fracture risk, and concludes that fat mass would be the main contributor to this relation. Controversely, other authors (6,15–17) sustain that a higher amount of lean mass would be beneficial and have a stronger influence on bone mass. Both statements have physiological plausibility. The fat tissue would represent the influence of neuroendocrine factors, as well as the metabolism of sex steroids (17) on bone density. The lean mass, on the other hand, would represent the mechanical and physical stimulation effect on bone tissue. Li and cols. (17), analysing perimenopausal women (average of 49.6 years), revealed that both fat mass and lean mass had positive relations with BMD of lumbar spine and femur. However, using multiple regression analysis, authors observed that only lean mass and ethnicity remained significant predictors of BMD of femoral neck. The lean mass was the only predictor of BMD of the total femur, explaining in 38% the BMD at this site, while fat mass was not a significant predictor of BMD in any of the analyzed sites.

The influence of total body mass and its different components on bone mass apears to vary according to age, gender and skeletal site of the studied populations (Table 4). In most studies, the lean mass apears to have a greater influence on bone density than fat mass, specialy at proximal femoral sites. In younger men, however, fat mass can have negative effects on bone mass (4). With aging, changes in body composition components induce an increment of fat mass followed by a decrement of lean mass. Considering postmenopausal American women, Chen and cols. (18) also observed that lean mass exerts a stronger influence on the BMD of differente bone sites.

**Table 4:** Description of published studies which investigated relationships between body compartments and bone density in different populations

Author	Country	Population	Age (years)	Lean mass	Fat mass	Additional finding
Chen and cols., 1997 (18)	USA	50 postmenopausal Caucasian women	> 65	Strongest determinant of bone mass, especially total bone mass and bone content		The increase in body mass showed significant association with the increase in bone mass
Ho-Pham and cols., 2010 (15)	Vietnam	210 postmenopausal women	50 to 85	Positive influence on spine and femur bone mass	Positive influence on spine and femur bone mass	
Zhu and cols., 2015 (19)	Australia	915 men and 1014 women	45 to 66	Predicted bone mass in both genders	Predicted bone mass in both genders	
Gjesdal and cols., 2008 (16)	Norway	2214 men and 2991 women	47 to 50 and 71 to75	Predicted bone mass in both genders	Predicted bone mass in both genders	
Cui and cols., 2007 (4)	South Korea	737 men and 867 women	19 to 80	**Younger:** positive influence on all bone mass sites	**Younger:** negative influence on all bone mass sites	Lean mass was considered an important predictor of bone mass; however, fat mass also positively contributed to bone mass in postmenopausal women and older men
				**Older:** positive influence on all bone mass sites	**Older:** positive influence on forearm and calcaneus bone mass	
				**Premenopausal:** positive influence with all bone sites	**Postmenopausal:** positive influence with all bone sites	
Gillette-Guyonnet and cols., 2000 (5)	France	129 healthy women	75 to 89	Positive influence on all bone mass sites	Positive influence on all bone mass sites	
Taaffe and cols., 2000 (20)	USA	54 women Non-Hispanic Caucasians and Mexican-Americans with BMI < 30 kg/m^2^	60 to 86	**Non Hispanic:** positive influence on bone mass of lumbar spine. **Mexican-Americans:** positive influence on bone mass of lumbar spine and trochanter	**Non Hispanic:** positive influence on femoral neck bone mass	

In a Korean rural population (19 to 80 years old, both genders), lean mass was an important determinant of BMD either for young and elderly population, but fat mass showed a dual effect. High fat mass showed negative influence on bone mass in younger, however, with positive influence in postmenopausal women and older men (4).

The physiological mechanisms that would explain this important correlation between bone mass and body mass are not completely defined yet. Some experimental studies suggest the existence of a bone remodeling central control that would work via neuropeptides and neurotransmitters, such as serotonin (21,22) in addition to adipokines, as leptin, which would connect fat tissue to bone metabolism (22). Karsenty (23) postulate that bone tissue has an endocrine role in the regulation of energy metabolism, especially decarboxylated osteocalcin, acting in the regulation of glucose homeostasis and insulin sensitivity (21–28).

It is well established that mechanical loading has a powerful anabolic effect on bone tissue, an effect coordinated by osteocytes (29). This can be confirmed by increased bone mass observed in athletes, when compared with sedentary controls (30). On the other hand, immobility and inactivity are considered great causes for low bone mass, risk of falls and fractures (31). Thus, lean mass measured by DXA can be interpreted as a marker of bone health, which means a greater mechanical load on the skeleton and would justify our findings. This gives better basis for the importance of encouraging physical activity to maintain muscle mass in preventing osteoporosis (32).

In conclusion, revious studies, as well as the present one, confirm that several variables contribute to the association between lean mass, fat mass and bone mass. Among these variables we highlight the treatment for osteoporosis, different ages and stages of life, gender and ethnicity. Our data revealed an important relationship between total body mass and all bone mass sites in postmenopausal women without osteoporosis treatment. However, in women being treated for osteoporosis these correlations lose their relevance. Among the different BC components, we found that lean mass was the one that presented the best correlation with bone mineral density, mainly on the proximal femur. In multiple variables model, when lean mass was corrected by the age of women without treatment for osteoporosis it explained about 30% of proximal femur bone mass. These results suggest that maintaining a healthy muscle mass can contribute to decrese the risk for osteoporosis. Results like ours also stimulate the search for mechanisms that explain this phenomenon, as well as the relevance of BC parameters on bone mass during treatment of osteoporosis.
